# Correlating Optical and Structural Properties of CO on Transition Metal Surfaces

**DOI:** 10.1021/acs.jpcc.4c07418

**Published:** 2025-03-03

**Authors:** Mai-Anh Ha, Dimitar Pashov, Mark van Schilfgaarde

**Affiliations:** † Computational Science Center, National Renewable Energy Laboratory, 15013 Denver West Parkway, Golden, Colorado 80401, United States; ‡ Department of Physics, King’s College London, Strand, London WC2R 2LS, U.K.; § Materials Chemical & Computational Science, National Renewable Energy Laboratory, 15013 Denver West Parkway, Golden, Colorado 80401, United States

## Abstract

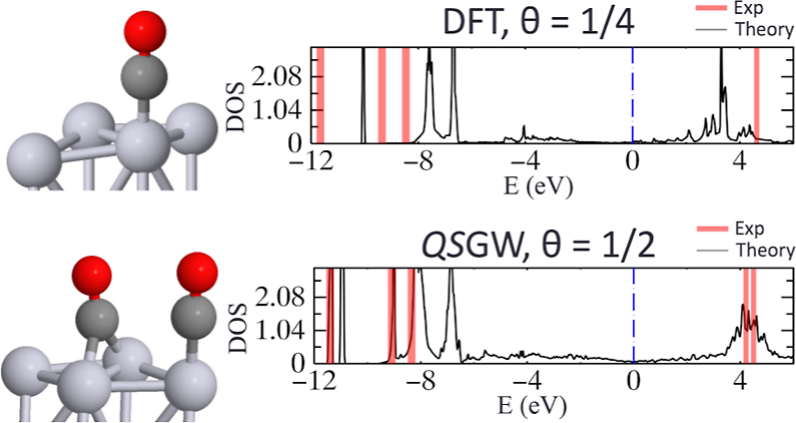

We present an optical study based on the quasiparticle self-consistent *GW* (QS**GW**) approximation combining structural information taken from density functional theory (DFT) to elucidate spectral features of CO adsorbed on Pt(111) and Cu(111). Optical information and structural arrangement of the adsorbed CO are correlated by varying both site positions and CO coverage as compared to experimental studies (θ = 1/4 to θ = 1/2). This enables us to resolve key spectral features of both occupied and unoccupied molecular states at various adsorbate coverages, comparing theory to experiment. Using experimental data as benchmarks, we show the theory compares well with available data. Its predictive power provides a new path to infer information about the structure of CO from optical information and can help to predict the presence of other little understood adsorbates such as an OCCO dimer that may be relevant to mechanistic pathways for reduction of CO_2_ to high value C_2_^+^ products. This new approach complements total energy calculations and also fills a void in DFT-based theory that is known to be an unreliable predictor of the energetics of CO on transition metal surfaces.

## 1 Introduction

CO is an important probe molecule in infrared spectroscopy,^1^ which describes local electrostatic and chemical environments in heterogeneous catalysis and in electrochemical characterization^2−4^ of transition metal (TM) active sites (CO-stripping voltammetry). Moreover, CO is a key reactant product in CO_2_ reduction to low-carbon fuels and high value products such as alcohols, acids, and hydrocarbons and in carbon sequestration for reducing greenhouse gases.^5,6^ However, our theoretical understanding of CO adsorbed on a TM surface is fragmented. This is largely because our main tool to model such systems—density functional theory (DFT) even with functionals tuned for catalysis—lacks sufficient fidelity to reliably describe either the total energy or spectral properties, as we show in some detail in this work. As one example, many functionals strongly overbind CO at the hollow site on TM surfaces.^7^ Such uncertainty hampers the informative and predictive nature of theoretical studies of CO_2_ reduction. How CO initially binds to the substrate is an essential component in the initial step for processes, such as CO_2_ splitting into CO* and O*. If already the site preference of the initial step of the reaction process cannot be resolved to the required accuracy, modeling the entire reaction process remains in doubt. That being said, even while we find DFT energies are not reliable, we will show that DFT predictions of the structure (bond lengths) are much less variable between functionals and align much better with available experimental data.

The present work focuses on characterizing adsorption through spectral properties: although they are related to total energy, spectral functions provide different kinds of information about the chemistry of the interface. As with the total energy, the uncertainty in DFT predictions is too large to make the theory predictive. Here, we use a self-consistent form of many-body perturbation theory (the quasiparticle self-consistent *GW* approximation—QS**GW**^8^), which has much higher fidelity than DFT, and also higher fidelity than the *GW* approximation based on DFT. QS**GW** describes energy bands for a wide range of materials rather well^9,10^ including many where the local density approximation (LDA) fails. QS**GW** contains physical effects found in other theories, such as LDA + *U*, hybrid functionals, self-interaction correction, and *GW* in a consistent manner without many of their drawbacks, such as partitioning of itinerant and localized electrons, adjustable parameters, and ambiguities in double counting. QS**GW** accurately captures valence d bandwidths, magnetic moments, and the positions of s- and d-bands in 3d metals.^9^ It has also been applied to the GW100 set of molecules and shown to yield very good ionization potentials.^11^ In a study of the Fe/MgO magnetic tunnel junction (a system with some similarities to the present work), it gave an excellent description of the transmission probability.^12^

The QS**GW** formalism can be derived from a standpoint of self-consistent perturbation theory, where the unperturbed Hamiltonian is an optimized noninteracting one.^10^ Ismail-Beigi showed that formalism can also be derived from a variational principle, though not the usual one.^13^ For insulators, QS**GW** provides rather satisfactory description of valence bands; conduction bands are also well described, although bandgaps are systematically overestimated—a feature which can be almost completely ameliorated by adding ladder diagrams to the polarizability, often even in strongly correlated systems.^14^ This demonstrates that the charge susceptibility, which generates the correlation part of the potential, is well described by QS**GW**. In metals, QS**GW** provides an excellent description of ARPES provided fluctuations in the spin channel are not too strong, e.g., in Fe.^9^ Questaal’s implementation of QS**GW** is described in detail by Pashov et al.^15^

For benchmarking adsorption of CO on TM surfaces, we take structures of the adsorbed molecule using DFT, and we perform QS**GW** calculations on them. We compare to photoemission (PE) data for CO on Cu(111)^16−18^ and Pt(111).^19,20^ Pt(111) systems remain the best characterized experimental studies of CO preferentially adsorbing on an atomic site, whereas Cu(111) is an experimentally relevant catalyst for electrochemical CO_2_ reduction.^5,21^ The theoretical insights to CO in reference to PE data will be a significant first step to enabling more accurate predictions of the mechanisms for CO_2_ reduction and may be especially relevant for electrochemical, photoactive, and plasmonic applications.^5,22,23^

### 1.1 Prior Theoretical Studies of the Energetics of CO Adsorption

Patra et al. pointed out that the hollow site preference on (111) extends to Rh, Pt, Cu, Au, and Ag for the LDA and PBEsol functionals, though this discrepancy with experiment can be mitigated in some cases, e.g., CO on Rh and Ag through use of the PBE and SCAN functionals.^7^ Similarly, the hybrid functionals PBE0 and HSE03 can correctly predict the atop site preference for Cu, Rh, but not for Pt.^24^ Moreover, the portion of Fock exchange in hybrid functionals is a freely chosen parameter, which can be adjusted to correspond to the experimental property of interest. While HSE captures the 5σ (HOMO) and 2π* (LUMO) features well for Pt(111), it overestimates the metal bandwidth.^25^ Lazić, et al. found that setting a cutoff distance for nonlocal interactions through the van der Waals density functional could recover an atop site preference, but only at coverages of θ = 1/12.^26^ Once their theoretical study reached a coverage of θ = 1/4, the difference between the atop and hollow sites was negligible.

In these theoretical studies, the long-standing puzzle of CO on TM surfaces is attributed to charge transfer between CO and the metal site, with the preferential hollow site resulting from the enhanced back-donation of the metal d-orbitals to CO’s 2π* orbital. Shifting the LUMO of CO’s 2π* orbital higher can potentially reorient the HOMO–LUMO gap to revert to the correct 5σ-metal interaction for the atop site.^25,27^ Studies by Gajdoš and Hafner as well as Patra et al. add a Hubbard *U* parameter to surmount difficulties of the PBE functional, but note that the determination of *U* is empirical.^7,27^ Olsen et al. successfully stabilized the site geometries of atomic > bridging > fcc for CO on Pt(111), reflecting experiment, by implementing a combination of atomic orbitals from Herman–Skillman-type calculations and Slater-type orbitals with basis set expansion up to QZMP with scalar relativistic and spin–orbit corrections.^28^ This study proposed that DFT may be capable of resolving the correct site geometry when scalar relativistic or a combination of scalar relativistic and spin–orbit corrections are implemented. Total energies calculated within the random phase approximation, using LDA eigenfunctions and eigenvalues, seem to fare much better.^25^ In the same work, densities of states were also presented and showed improvement relative to DFT.

### 1.2 Role of Coverage Dependence

In our present study, we consider surface-CO coverages reflective of experimental conditions, where coverage θ can range from 0.17 to 0.5, depending on the study.^16−20^ This can necessarily alter the site geometries of CO molecules with a low coverage, which consists primarily atop CO sites, and coverages of 0.33–0.5, which achieve mixtures of atop, bridge, and hollow-site CO.^29−32^ Nekrylova et al.’s vibrational and thermal desorption spectroscopic study of CO on Pt(111) at 20 K found that the hollow-site CO’s infrared (IR) band of 1736 cm^–1^ appeared at θ = 0.35 and increased in intensity as the coverage reached θ = 1.0.^32,33^ With the advent of in situ scanning tunneling microscopy (STM), the ratio and position of atomic, bridging, and hollow sites can be precisely identified, highlighting the importance of CO–CO lateral interactions.^34,35^

Moreover, while both in-situ STM and IR-absorption spectroscopy (IRAS) can agree on the appearance of different sites at specific coverages, the ratio of the different kinds of occupied sites can vary considerably as demonstrated in a study of CO on Pt(111) by Villegas and Weaver.^34^ At θ = 0.75, STM images found a ratio of atomic:hollow site occupation of 1:2 per unit cell, while integration of corresponding IRAS data yielded 2:1. Since STM is likely the more reliable measure, the discrepancy between these experiments warrants some caution in inferring coverage from IR data. The difference between the two experiments was attributed to the dynamic dipole coupling effects related to CO–CO interactions, but it also suggests that there can be some ambiguity in the exact occupation of specific sites when analyzing IRAS data. Moreover, the richness of the experimental data indicates that theoretical models should take into account information about the coverage and distribution of sites in the actual experimental samples. We consider atomic, bridging, and fcc hollow CO sites at θ = 1/4 coverage and combinations of them which correspond to experiments at θ = 1/2 coverage. This allowed our theoretical model to closely match the coverages present during the experimental conditions of different PE studies.^16−20^ In this work, we elucidate how QS**GW**’s ability to match PE data with high fidelity makes it possible to resolve the unique features related to different CO site occupations.

## 2 Methods

The (111) surface was cut from a bulk unit cell of experimental lattice constant 3.9231 Å for Pt and 3.61496 Å for Cu.^36^ For CO adsorption on Pt(111) and Cu(111), we utilized a four-layer surface cell with four surface metal atoms exposed; for coadsorption of two CO molecules on Pt(111) and Cu(111), we utilized a six-layer surface cell with four surface metal atoms exposed. The Monkhorst–Pack *k*-point grid was 12 × 12 × 1 centered at the Gamma due to the hexagonal cell used for the (111) surface. A vacuum gap of double or more the height of the surface was incorporated in order to prevent spurious interactions between the periodic images. Subsequently, the calculated adsorption energies utilized the following equation
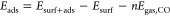
where *E*_surf+ads_ is the total energy of the surface with adsorbate; *E*_surf_ is the total energy of the clean surface without adsorbate; *E*_gas,CO_ is the total energy of the gas phase CO; and *n* = 1 and 2 as the number of CO gas molecules. Relative adsorption energies (Δ*E*_ads_) refer the energies of different isomers with respect to the global minimum structure, e.g. (Δ*E*_ads_ = *E*_ads,isomer_ – *E*_glob_). Postprocessing of isomers was performed, extracting electronic information for bonding analysis such as charge-transfer via the Bader charge algorithm and orbital overlap from projected density of states (PDOS).^37−41^ For geometric relaxation, the plane-wave DFT code, the Vienna Ab initio Simulation Package (VASP 5.4.4)^42−45^ was utilized and Supporting Information Tables S1–S4 summarize metal-C and C–O bond distances of these calculations. The plane-waves basis set was expanded to a kinetic energy cutoff of 520 eV, incorporating optimization of geometries to a convergence criteria of 10^–6^ (10^–5^) eV for electronic (geometric) relaxation and Gaussian smearing to account for the complexity of metal–adsorbate properties. A dense *k*-point mesh of 12 × 12 × 1 was implemented. For subsequent quasiparticle, self-consistent *GW*-level calculations, Bayesian error estimation functional with van der Waals correlation (BEEF–vdW) geometries were computed with an 8 × 8 × 1 *k*-point mesh for the one-body part and a 4 × 4 × 1 *k*-point mesh for the two-body part. The cutoffs to the *GW* basis sets were set at 5.0 a.u. for CO on Cu(111) calculations and 4.5 a.u. for CO on Pt(111) calculations. The self-consistency cycle was iterated until the change in self-energy fell below 1 × 10^–5^. QS**GW** calculations were performed with the Questaal package.^15^ Computing parameters and time for these QS**GW** calculations on NERSC’s Perlmutter cluster for CO on Pt(111) is summarized in Supporting Information Table S5 to give readers a sampling of Questaal’s performance.

## 3 Results and Discussion

We generated structures with relaxed, optimized coordinates for CO adsorbed onto a surface using three kinds of density functionals: LDA,^46^ Perdew–Burke–Ernzerhof (PBE),^47^ and BEEF-vdW.^48^ The BEEF-vdW functional was designed particularly to target heterogeneous catalysis with consideration for chemical reactivity (bond breaking and forming), the solid-state physics of the surface, and van der Waals forces. Probably for that reason BEEF-vdW tested better than the other functionals for CO-related reactions, often resulting in only a ∼0.1 eV deviation from experiment for CO oxidation (2CO + O_2_ → 2CO_2_, the reverse reaction to CO_2_ reduction), methanol formation (CO + 2H_2_ → CH_3_OH + H_2_O), and ethanol production (CH_3_ + CO + H_2_ → CH_3_CH_2_OH).

### 3.1 CO on Pt(111)

DFT has a well-known tendency to underestimate the splitting between HOMO and LUMO levels, and further, it tends to misalign heterogeneous orbitals, notably s, p states relative to TM d states. Both tendencies are seen here, with the occupied 4σ, 5σ, and 1π states too shallow and the unoccupied 2π* state too close to the Fermi energy. Both issues are connected with the DFT construction, namely, that the potential is the same for all electrons. Hybrid functionals such as HSE add nonlocal character missing in a pure density-functional, and energy levels are generally better. However, the amount of nonlocal character cannot be universal, since it depends on the dielectric response of the system, as has been shown by several authors; see Vidal et al.^49^ In the CO/TM systems studied here, the screening itself is highly heterogeneous. For high fidelity, a theory must take into account the effects of heterogeneous screening, and also the feedback between energy levels and the dielectric response, and finally the corrections to one-body (Hartree) parts of the potential from modification of eigenfunctions by a higher fidelity potential.^50^

As regards ground-state properties, the failure of LDA and PBE functionals to stabilize the CO on the atop site is well-known. A considerable number of studies have delved into the short-comings of DFT and the possibilities of hybrid functionals such as HSE, RPA, and Hubbard *U* corrections to resolve some aspects of the HOMO–LUMO gap for CO on transition metals.^7,24,25,27^ These studies hypothesize that the *interaction* between CO’s orbitals to the TM’s d-orbitals lead to enhanced back-donation to CO’s 2π* orbital that preferentially stabilizes the hollow site instead of the directional 5σ orbital for atop site adsorption. Furthermore, DFT often shifts both the 5σ and 2π* states closer to the Fermi energy, resulting in an overestimation of the binding energy. Nevertheless, where experiments are available, we can compare the relative energies of the three adsorption sites. Additionally, we present results for two other ground state properties: the site-resolved partial charges using a Bader analysis, and bond distances across the different functionals (Supporting Information Table S1). In contrast to the total energy, bond lengths are remarkably uniform across functionals and in good agreement with experiments. This supports our argument that DFT is sufficient to determine structure, if not total energy, and subsequently may not capture accurately the spectral features related to photoelectron, photoemission (PE) experiments.

**Figure 1:**
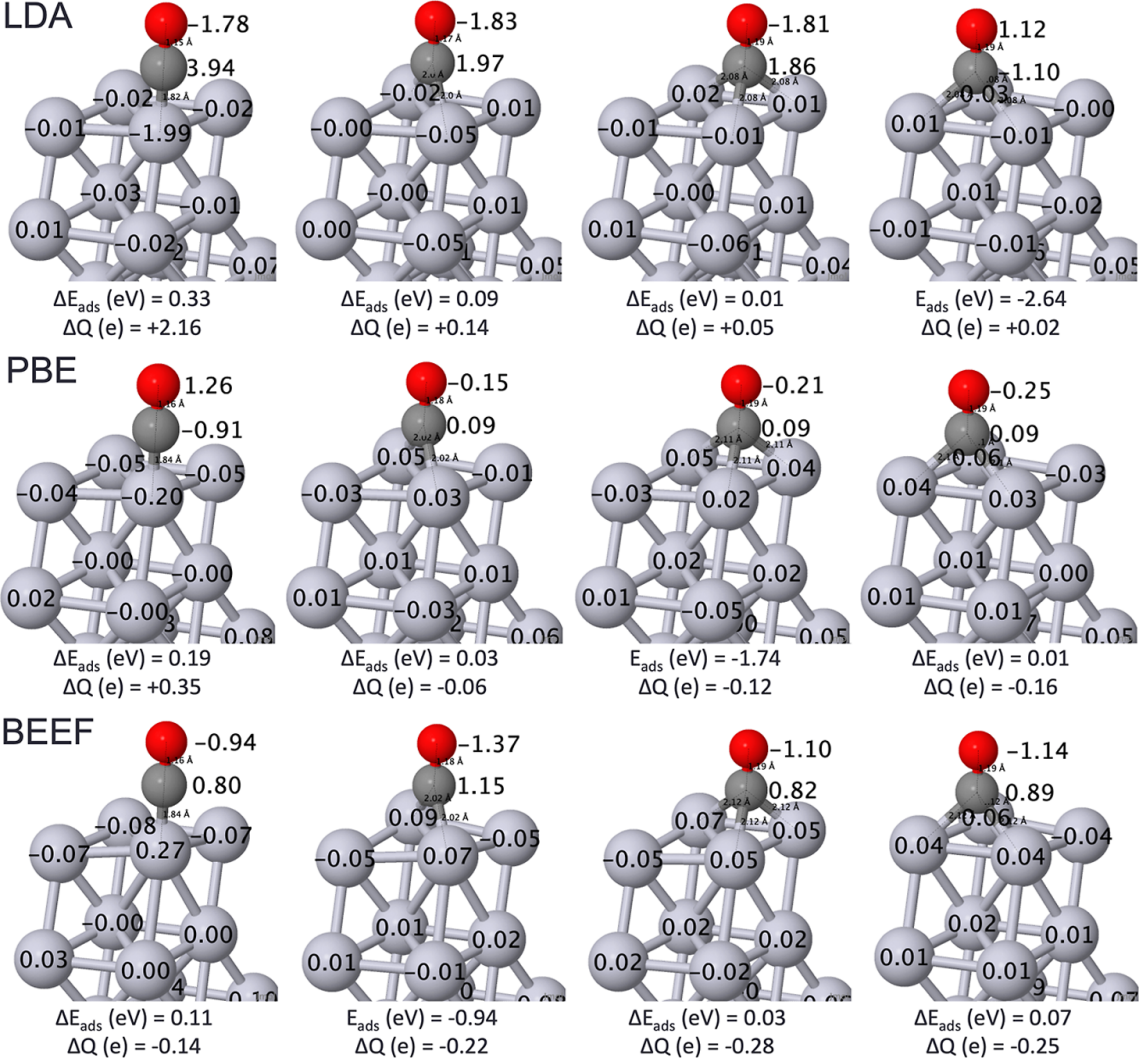
Visualization of CO’s four unique adsorption sites of atomic, bridging, fcc hollow, and hcp hollow sites (from left to right) with adsorption energies (*E*_ads_, eV), relative adsorption energies (**Δ***E*_ads_, eV), Bader charges (**Δ***Q*, e), and bond lengths (Å). Bond lengths are compared to experimental values in Supporting Information Table S1. Pt—light gray, C—dark gray, and O—red. Adapted from ref (51) and available under a CC-BY 4.0 license. Copyright 2024 Ha et al.

Regarding Bader charge analysis, the most important finding is that partial charges vary wildly across functionals (Figure 1).^39,40^ For the LDA functional, the CO was positively charged for all of the adsorption sites; the PBE and BEEF-vdW functionals resulted in negatively charged CO for all of the sites. Not only did the LDA destabilize the atomic site considerably compared to the hollow site, but it also exhibited the greatest polarity within the CO molecule. Indeed, the CO molecule became circa +2e and the Pt atom below it −2e. The magnitude of these interactions decreased from LDA to PBE to BEEF-vdW calculations.

By comparison, the bridging and hollow sites were inconsistent in the magnitude of the charge transfer across the Pt, C, and O atoms. Since the energy is a function of the charge, it is not surprising that the total energies based on DFT are unreliable. Forces are also controlled by charge (the density at the nucleus), yet surprisingly, the bond distances between the Pt–C and C–O bonds remain similar across the different functionals, with a relative difference of only 0.04 Å. Intensity analysis of measured *I–V* curves in a dynamical low-energy electron diffraction study provided considerable detail to surface CO–Pt (111) bonds.^30^ Indeed, the experimental uncertainty of the C–O bond 1.15 ± 0.05 Å is broader than the variation in C–O bond lengths of all three site types (1.15–1.16 Å for atop CO, 1.17–1.18 Å for bridge CO, and 1.19 Å for hollow CO). A similar trend occurs for the Pt–C bond, wherein all three functionals can reproduce bond lengths within the uncertainties of the experiment.

Table 1 compares the total energy for the three absorption sites, along with measured values taken from He scattering for desorption of CO and IRAS-LEED.^29,52,54^ Notably, the functionals differ considerably in both their reaction energies and their relative changes in energies across the three adsorption sites. BEEF-vdW performed best for relative energies, with Δ*E* ranging between 0.00 and 0.11 eV, compared to measured values of Δ*E* = 0.00 – 0.11 eV.^54^ This is a marked improvement over that of LDA (Δ*E* = 0.00 – 0.33 eV) and PBE (Δ*E* = 0.00 – 0.19 eV). Even for BEEF-vdW, some discrepancies with experiment are seen. It finds the bridging site to be more stable than the atomic site by 0.11 eV, while experimentally, this difference is −0.07 eV.^52,54^ Subsequent QS**GW** calculations were evaluated for structures derived from the BEEF-vdW functional since it performs best. From QS**GW**, we can evaluate PDOS to compare to experimental PE data.^19,20^ PDOS allows us to elucidate the s-, p-, and d-orbital contributions by Pt, O, and C and correlate their contributions to the σ- and π-levels determined in the experiment.

**Table 1:** Adsorption Energy (*E*_ads_) and Relative Adsorption Energies (Δ*E*_ads_) to the Global Minimum on Pt(111) at θ = 1/4

	Exp, Δ*E*_ads_ (eV)	LDA, Δ*E*_ads_ (eV)	PBE, Δ*E*_ads_ (eV)	BEEF, Δ*E*_ads_ (eV)
atomic	0.00 (−1.38),^52^ (−1.43)^53^	0.33 (−2.31)	0.19 (−1.55)	0.11 (−0.83)
bridge	0.07,^54^ 0.02^53^	0.09	0.03	0.00
FCC hollow	0.11^29^	0.01	0.00	0.03
HCP hollow		0.00	0.01	0.07
*E*_ads,glob_ (eV)	–1.38	–2.64	–1.74	–0.94
Exp (eV), *E*_atom→bridge_	0.156 ± 0.029^31^			
Exp (eV), *E*_bridge→atom_	0.08,^54^ 0.091 ± 0.019^31^			

Experimental values of CO-derived σ- and π-levels below the Fermi energy *E*_F_ on Pt(111) are reported for θ = 1/2 coverage, with an equal amount of atop- and bridge-CO sites reported to be occupied.^19,30^ More in-depth IRAS studies observe that due to the 0.04 eV energy difference between the bridging and hollow sites, Boltzmann populations factor in the occupation of the hollow sites. Even at temperatures as low as 20 K, the hollow site’s IR band appears at θ = 0.35.^32,33^ For occupied σ and π CO states, Tsilimis et al. performed an ultraviolet photoemission spectroscopy study at 150 K, while Anazawa et al. determined the position of the unoccupied LUMO 2π* states from a two-photon PE (2PPE) study at <120 K.^19,20^ While atomic and bridging sites should be dominant, some non-negligible percentage of CO that occupies hollow sites is also likely.

**Figure 2:**
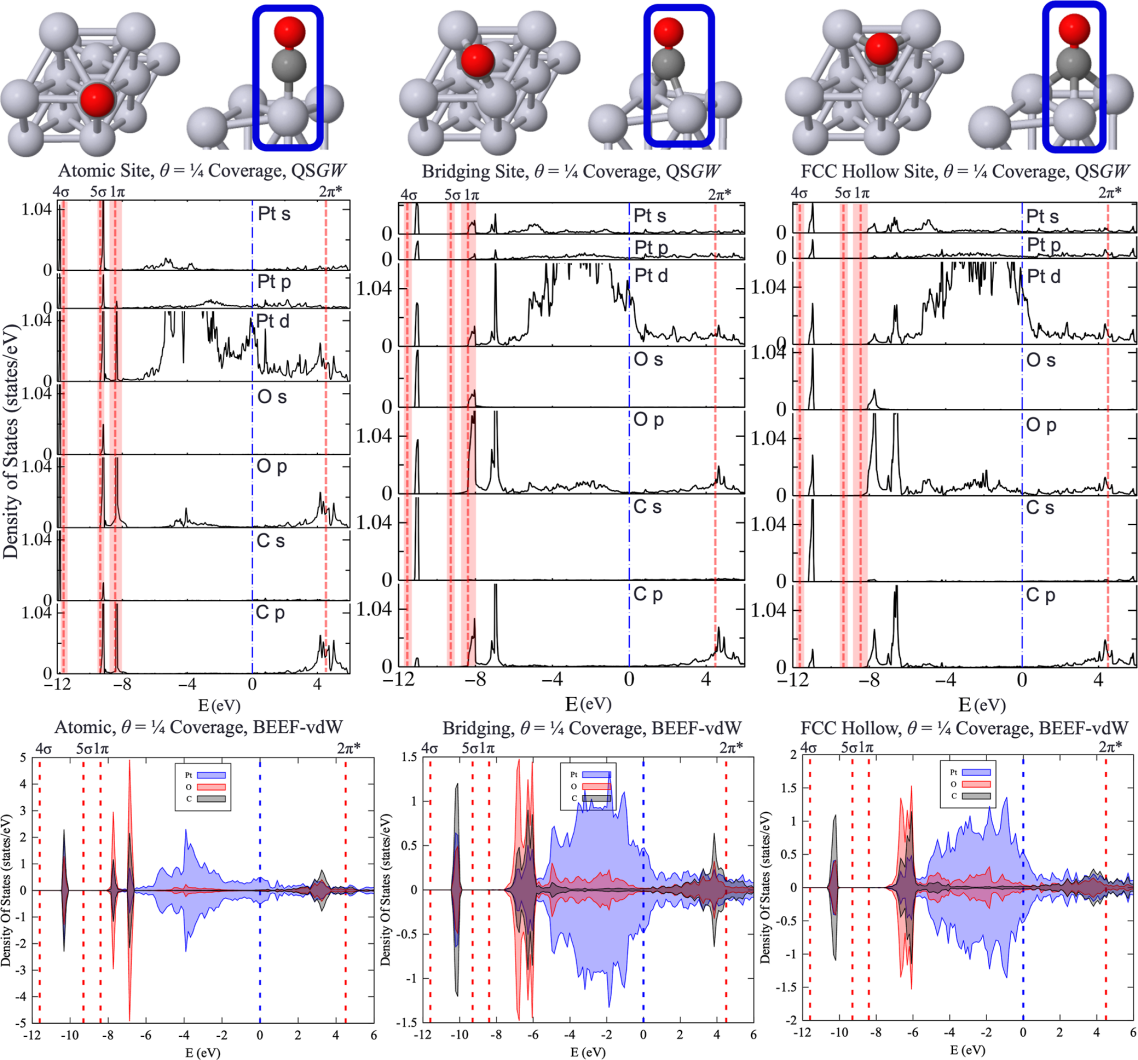
Theoretical PDOS, with dotted-red lines overlaid that reflect the molecular levels extracted from PE data. (**TOP**) Converged QS*GW* results from Questaal; (**BOTTOM**) DFT results from VASP. Data below the Fermi energy (*E*_F_) are taken from Tsilimis et al. (4σ at *E*_B_ = −11.6 ± 0.2 eV, 5σ at −9.3 ± 0.2 eV, and 1π at −8.4 ± 0.4),^19^ while the above *E*_F_ data were taken from a two-photon experiment from Anazawa et al. (LUMO state for the atomic site at 4.5 eV, <0.17 ML).^20^ Pink shaded areas reflect uncertainties reported in the measurements. All six DOS plots use the same experimental data. The abscissa was shifted to place the Fermi energy at 0. Adapted from ref (51) and available under a CC-BY 4.0 license. Copyright 2024 Ha et al.

As a first step, we calculated the partial DOS for all three configurations (atop, bridge, and fcc hollow) at θ = 1/4 coverage. Figure 2 shows the result together with levels extracted from PE measurements^19^ overlaid in red. As the PE measurements were taken for θ = 1/2 samples, they are not directly comparable to the calculated results. The comparison is nevertheless useful because it demonstrates that both occupied states and the LUMO 2π* state are sensitive to configuration, and therefore, the optical data can serve as a useful structural probe. For PE data,^19^ the authors report equal occupation of atop and bridge CO sites. If we accept this to be correct, the closest comparison to the experiment would be to average the calculated PDOS in the left and center panels in equal measure. On the other hand, if the total energies of Table 1 are accepted, the relative populations of the atomic and bridging site will differ by a Boltzmann factor, exp[−Δ*E*/*k*_B_*T*]. Taking into account the large uncertainty in Δ*E* (Table 1), the Boltzmann factor ranges between 0.21 for Δ*E* = 0.02 to 0.004 eV for Δ*E* = 0.07 eV. It is very likely, therefore, that the atomic configuration is much more populous than the bridging one.

This is consistent with QS**GW** calculations, which align very well with PE data for the atop site and much less so for the other configurations. Olsen et al. suggested that spin–orbit coupling might be a contributing factor to the accuracy of CO on TM surfaces.^28^ In Supporting Information Figure S1, a comparison of the PDOS from QS**GW** calculations without and with spin–orbit coupling is provided for atop CO on Pt(111) at θ = 1/4, reproducing the same peaks with negligible differences in magnitude. Spin–orbit coupling is computed as part of the single-particle Hamiltonian after QS**GW** self-energy is made. More properly, the effect on the self-energy should be included as well. However, we have found the effect to be quite small with a mild exception for the very heavy element Pb. We have a means to feed the SO coupling into the self-energy (see the appendix in Azarhoosh et al.),^55^ but even for Pb, it mainly affected the Pb p orbital. Based on Supporting Information Figure S1, we do not think the effect is significant here.

The PDOS was plotted for the different sites, showcasing that each site may contribute to the experiment within the uncertainty of the observed σ- and π-levels (Figure 2—**TOP**). The atomic site contributes the most intense peaks to the 5σ- (containing hybridization from Pt, O, and C s-, p-, d-orbitals) and 1π-levels (primarily Pt d-orbitals and C, O p-orbitals) of experimental PE data,^19^ whereas the 4σ-level may represent contributions from all three sites. While the atomic site shifts the 4σ band away from *E*_F_, the bridging and hollow sites shift the 4σ band closer to it. Therefore, at higher coverages, the mixing of the bands may shift closer to experimental σ- and π-levels below the *E*_F_. For the unoccupied LUMO 2π* state, the 2-photon study of Anazawa et al.^20^ places it at *E*_F_ + 4.5 eV at <0.17 ML coverage, with a second peak at 4.2 eV rising in prominence as bridging sites become occupied, with coverage increasing to θ = 1/2. QS**GW** predicts that all three sites can contribute to the 2π* peak at 4.5 eV, so this state cannot be used to discriminate the structure.

For comparison, PDOS from DFT results utilizing the BEEF-vdW calculations is displayed in Figure 2**(BOTTOM)** for the three geometries (atomic, bridging, and fcc hollow) of CO on Pt(111), finding similar features to Patra et al. In the PBE + *U* study by Patra et al., the atomic site’s most prominent peak was near *E*_F_ + 3 eV, with a small peak near *E*_F_ + 4 eV.^7^ On the other hand, they found prominent peaks close to E_F_ + 4 eV for the fcc hollow and hcp hollow sites, similar to QS**GW** results. While BEEF-vdW can shift the adsorption energy and the relative stability of these site geometries, it cannot resolve the density of state features to the level of accuracy displayed by self-consistent QS**GW**. BEEF-vdW’s 2π* states above the Fermi energy are shifted by 0.5–1.0 eV from experiment and the 4σ, 5σ, and 1π states below the Fermi energy are shifted by ca. 2 eV from the experiment.

**Figure 3:**
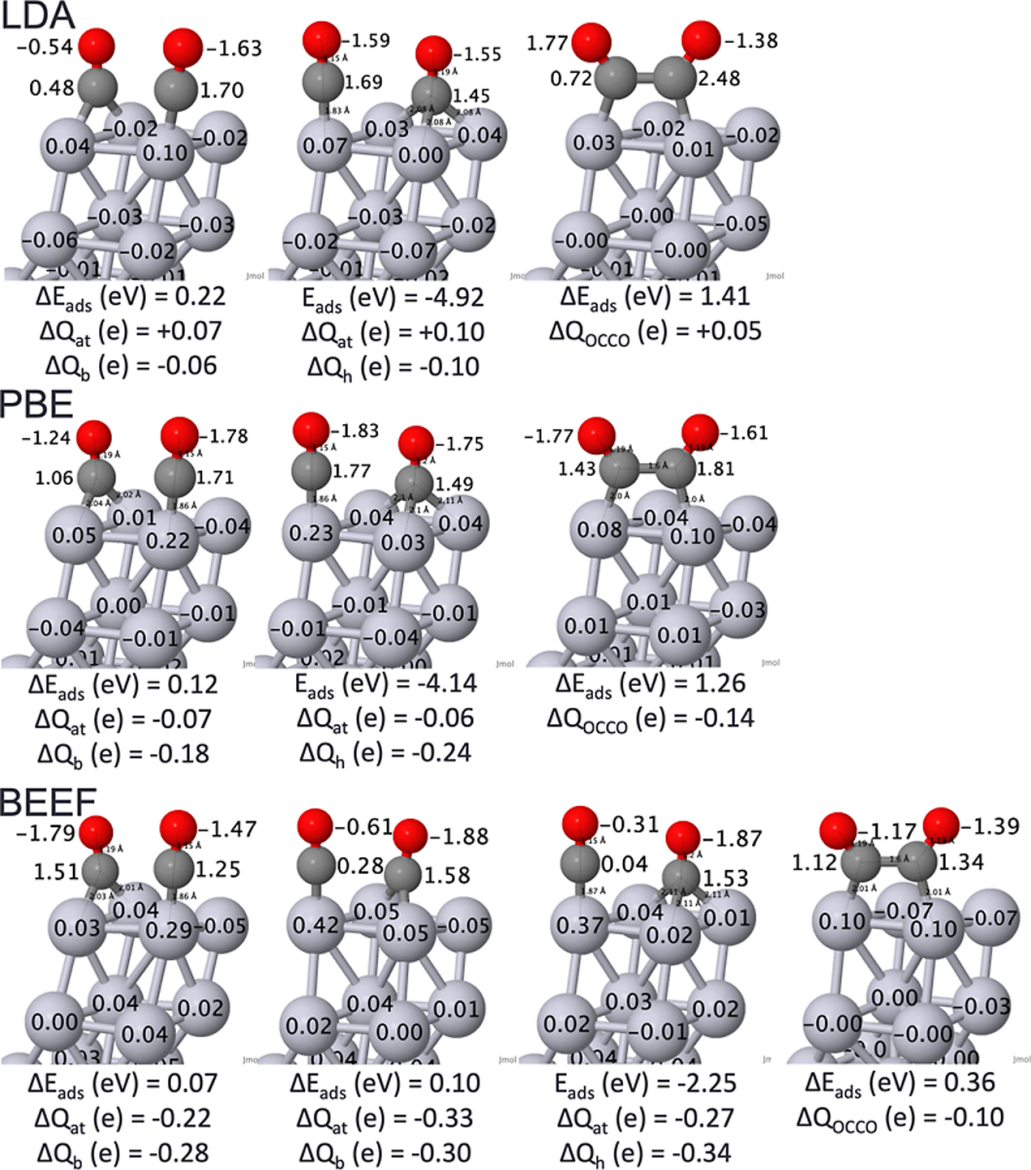
Visualization of unique 2CO/Pt(111) of atomic + bridging, atomic + fcc hollow, and OCCO dimer with adsorption energies (*E*_ads_, eV), relative adsorption energies (**Δ***E*_ads_, eV), Bader charges (**Δ***Q*, e), and bond lengths (Å). Pt—light gray, C—dark gray, and O—red. Supporting Information Table S2 contains bond lengths. Adapted from ref (51) and available under a CC-BY 4.0 license. Copyright 2024 Ha et al.

Next, we consider the θ = 1/2 coverage by coadsorbing a pair of CO molecules on the surface, which is better reflective of the experimental configurations. We relaxed the atomic + bridging and atomic + fcc hollow pairs with the LDA, PBE, and BEEF-vdW functionals, as we did for the θ = 1/4 configurations. In the course of relaxation, a new configuration (the OCCO dimer) emerged spontaneously. Figure 3 presents cartoons of the resulting structures along with Bader charges and energetics. The relative energies of these isomers can be sensitive to the magnitude of charge transfer between atoms: for the BEEF-vdW functional, there are two minima for the atomic + bridging configuration with a relative energy of 0.03 eV between them. Geometrically, they are nearly identical, but the slightly more stable isomer features more extreme positive (negative) charges on the C (O) atoms for the CO on an atop site. Therefore, they may be considered two unique minima due to the degree of charge transfer between the C–O atoms. Bond lengths are compared to experimental data in Supporting Information Table S2.

The atomic + fcc hollow configuration remains the most stable across the different functionals, although the relative energy of the coadsorbed atomic and bridging site from the atomic and fcc hollow site decreases from the LDA to the PBE to the BEEF-vdW functional. Also, Bader charges in the 2-CO geometries fluctuate less across functionals with the majority of the CO molecules exhibiting a slight negative charge. The C–O and Pt–C bond lengths of these coadsorbed geometries reproduced similar values to those found at θ = 1/4 coverage and therefore complement experiment (Supporting Information Table S2). While LDA and PBE functionals found the OCCO dimer to be a 1 eV higher than other coadsorbed geometries; this difference was smaller with BEEF-vdW with only 0.36 eV relative to the atomic + fcc hollow site. This highlights the dependence of the functional on the relative energy of a possibly catalytically relevant species. A DFT study exploring CO dissociation routes on Pt and Pt–Sn also found the OCCO dimer, but determined the high formation barrier to be an energetically unfavorable mechanism.^56^ However, this conclusion may change, as it was predicated on a barrier that is likely too large. As observed in Figures 1 and 3, the energies of various minima can differ substantially, and BEEF-vdW has thus far drawn closest to the relative energies in experiments of the different sites (see Table 1). Therefore, there is some ambiguity on the energetic possibility of the OCCO dimer.

**Figure 4:**
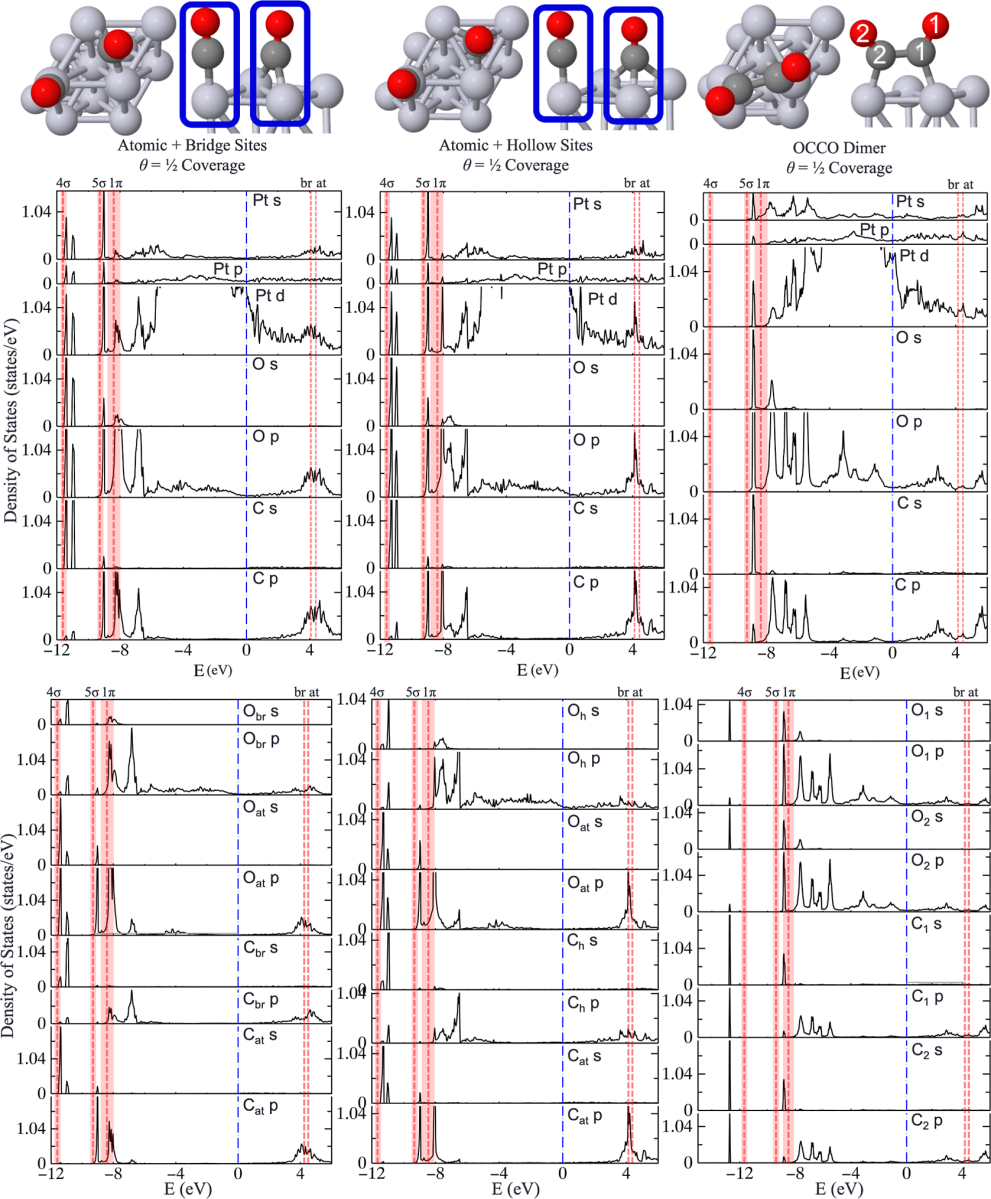
PDOS of 2CO/Pt(111) from QS*GW* calculations in geometries generated by BEEF-vdW: (**TOP**) Elemental analysis of Pt, C, and O orbital projections from the Pt–C–O atoms outlined in blue; (**BOTTOM**) atomic contributions of each C and O with subscripts indicating the specific atom to orbital contribution at—atomic, br—bridging, and h—hollow, and for the OCCO dimer, numerical subscripts 1 and 2 were utilized to differentiate C and O atoms. Dotted red lines depict PE peaks (4σ at *E*_F_ – 11.6 ± 0.2 eV, 5σ at *E*_F_ – 9.3 ± 0.2 eV, and 1π at *E*_F_ – 8.4 ± 0.4)^15^ and two-photon PE data for the 2π* LUMO (at *E*_F_ + 4.5 eV and *E*_F_ + 4.2 eV for atomic site and bridging site, respectively).^16^ Pink shaded areas reflect uncertainties reported in the measurements with abscissa shifted to place the Fermi energy at 0. Adapted from ref (51) and available under a CC-BY 4.0 license. Copyright 2024 Ha et al.

In Figure 4**(TOP)**, we visualize the PDOS of the coadsorbed atomic + bridging sites, atomic + fcc hollow sites, and the OCCO dimer. Compared to Figure 2, the influence of CO–CO on the position of the molecular levels can be seen. All three levels (4σ, 5σ, and 1π) below *E*_F_ align very well with respect to experiment. This provides the best benchmark of the theory, as the CO configurations are the closest to the experimental ones. The 4σ contains strong contributions from the Pt s- and d-orbitals, O s- and p-orbitals, and C s-orbital. For the 2π*, a smearing effect occurs within the 4.2–4.5 eV range found by Anazawa et al. for the bridging and atomic sites. In contrast, the PDOS for the atomic + fcc hollow site exhibits an intense peak at 4.2 eV with a small shoulder at 4.5 eV above *E*_F_. This suggests that interactions between a CO molecule on an atop site and a CO molecule on a hollow site may have also contributed to the 4.2 eV peak observed in the 2PPE experiments by Anazawa et al.

In addition to the elemental analysis provided in Figure 4**(TOP)**, we provide a detailed decomposition of the CO–CO s*-*, p-contributions to the experimental peaks in Figure 4**(BOTTOM)**. This allows us to probe the contribution of the atomic, bridging, and hollow CO molecules to experimental PE spectra, elucidating that the 4σ peak primarily arises from the shift of the atop site’s C’s s-orbital and O’s s-, p-orbitals toward the Fermi energy to match *E*_F_ – 11.6 eV for both atomic + bridge sites spectra and atomic + hollow sites spectra; the 5σ peak features most prominently the atop site’s C’s p-orbital and O’s s-, p-orbitals for both atomic + bridge sites spectra and atomic + hollow sites spectra; and 1π brings in p-orbital contributions from both the atomic + bridging or atomic + hollow sites’ C and O atoms. For the spectral features above *E*_F,_ this allows us to pinpoint that CO–CO interactions result in the *atop site*’s orbital contributions shifting to match experiment; this features significantly in the atomic + hollow site’s interactions and remains more delocalized for the atomic + bridging sites’ interactions. For the OCCO dimer, the contributions are fairly symmetric, with little differentiation in spectral features between C_1_ to C_2_ or O_1_ to O_2_.

As noted in numerous IRAS studies, the hollow site appears at circa θ = 0.3 and differs in energy from the bridging site by only 0.04 eV.^29,33^ Much of the focus of previous theoretical studies was on first achieving the correct adsorption energy and site geometry and secondarily achieving the HOMO–LUMO gap. By also considering the θ-dependence of the 4σ, 5σ, and 1π levels and the LUMO 2π*, we can directly connect to the experiment. Moreover, the benchmark described here establishes that QS**GW** is able to accurately predict these levels. Assuming a similar accuracy for other interfaces, this approach’s predictive power provides a powerful complement to the optical experiments. It provides a one-to-one correspondence between spectra and structure and can aid in the search for particular structures via PE experiments. The OCCO dimer provides an excellent illustration of this. For Cu substrates, it is thought to be an important intermediate state in the catalysis process.^57^ According to QS**GW**, its signature is markedly different from the atomic + bridging sites or atomic + fcc hollow sites, especially the shift in the 4σ state and the splitting of the 2π* peak to two states near 3 and 5 eV.

### 3.2 CO on Cu(111)

**Figure 5:**
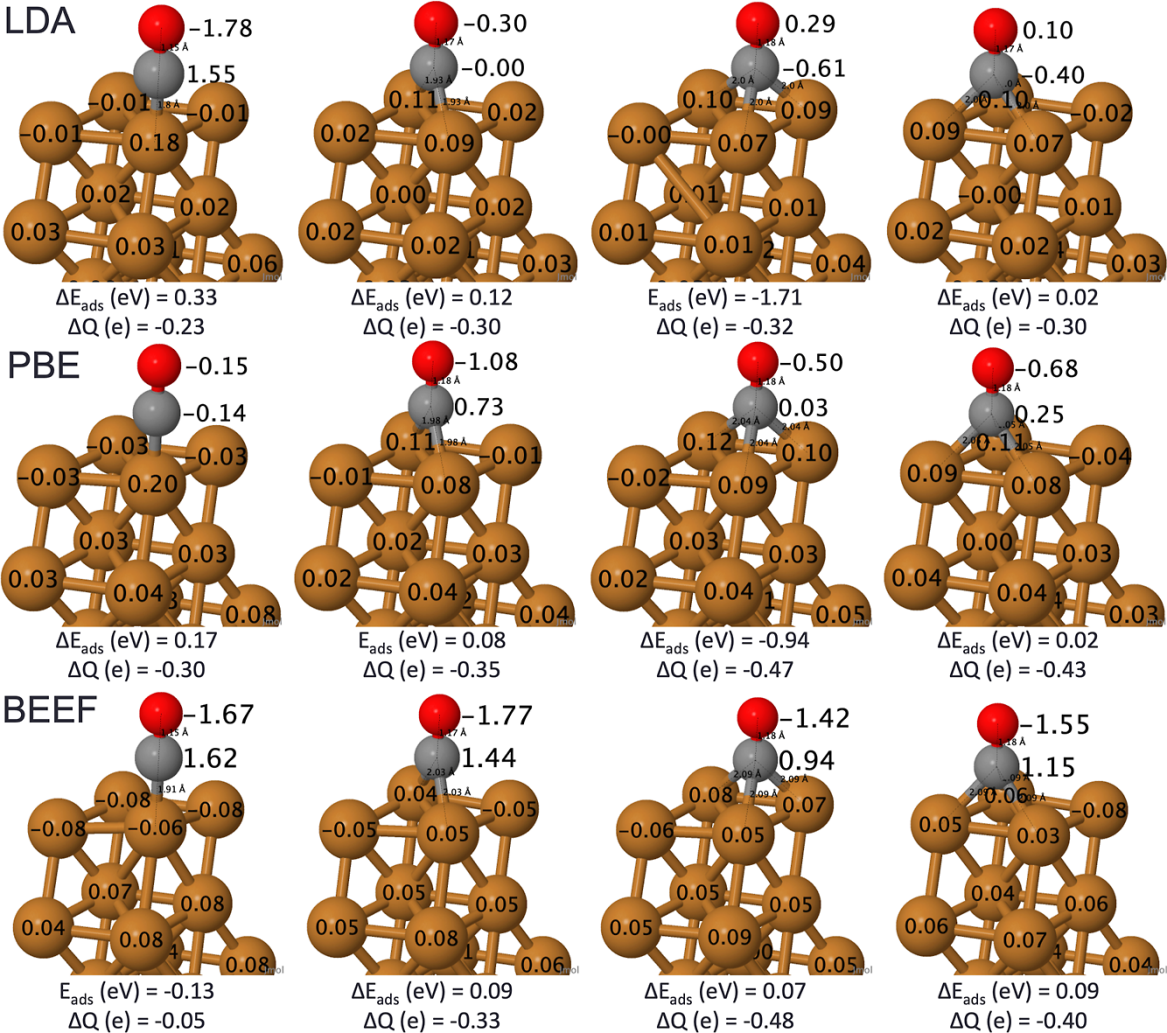
Visualization of CO/Cu(111) of atomic, bridging, fcc hollow, and hcp hollow sites (from left to right) with adsorption energies (*E*_ads_, eV), relative adsorption energies (**Δ***E*_ads_, eV), Bader charges (**Δ***Q*, e), and bond lengths (Å, details in Supporting Information Table S3). Cu—dark orange, C—dark gray, and O—red. Adapted from ref (51) and available under a CC-BY 4.0 license. Copyright 2024 Ha et al.

We now turn to Cu(111), a catalytically relevant material for CO_2_ reduction. Less experimental data is available for the Cu(111) case. This is most often attributed to the weak binding of CO (−0.52 eV), which decreases with increasing coverage of CO to −0.39 eV (Table 2). In an IRAS study comparing atomic, bridging, and hollow sites, Hayden et al. found only around 20 cm^–1^ difference in the IR bands between the bridging and hollow sites.^29^ They concluded that the energetic potential between the two states is shallower than the corresponding 0.04 eV difference on Pt(111) (see Table 2). In Table 2 and Supporting Information Table S3, we summarize the DFT-level energies and bond lengths as compared to the experiment; in Figure 5, these results are visualized. Unsurprisingly, the LDA functional resulted in the strongest binding energy, followed by the PBE functional. Regardless of the functional, Bader charges found the CO to be slightly negative, ranging from −0.05 to −0.48 *e*. While C–O bond (1.15–1.18 Å) remained similar across the different functionals, the Cu–C bond (1.80–1.91 Å) varied more significantly than the Pt–C bonds. The BEEF-vdW functional underestimated the experimental adsorption energy, but predicted a Cu–C bond (1.91 Å) matching experiment^59^ and correctly predicted the most stable site to be CO atop a Cu. Therefore, we used BEEF-vdW geometries in subsequent self-consistent *GW* calculations.

**Table 2:** Adsorption Energy (*E*_ads_) and Relative Adsorption Energies (Δ*E*_ads_) to the Global Minimum on Cu(111) at θ = 1/4^a^

	LDA Δ*E*_ads_ (eV)	PBE Δ*E*_ads_ (eV)	BEEF Δ*E*_ads_ (eV)
Atomic	0.33 (−1.38)	0.17 (−0.77)	0.00 (−0.13)
Bridging	0.12	0.08	0.09
FCC hollow	0.00	0.00	0.07
HCP hollow	0.02	0.02	0.09
*E*_ads,glob_ (eV)	–1.71	–0.94	–0.13
Exp, *E*_ads_ (eV)	–0.52 (θ ≤ 1/3), −0.39 (θ > 1/3)^58^		

a In parentheses, we include the adsorption energy of the atomic site to more directly compare to experiment values. Only BEEF-vdW successfully stabilized the atomic site as the global minimum; LDA and PBE overbind and stabilize the hollow site over the atomic site.

**Figure 6:**
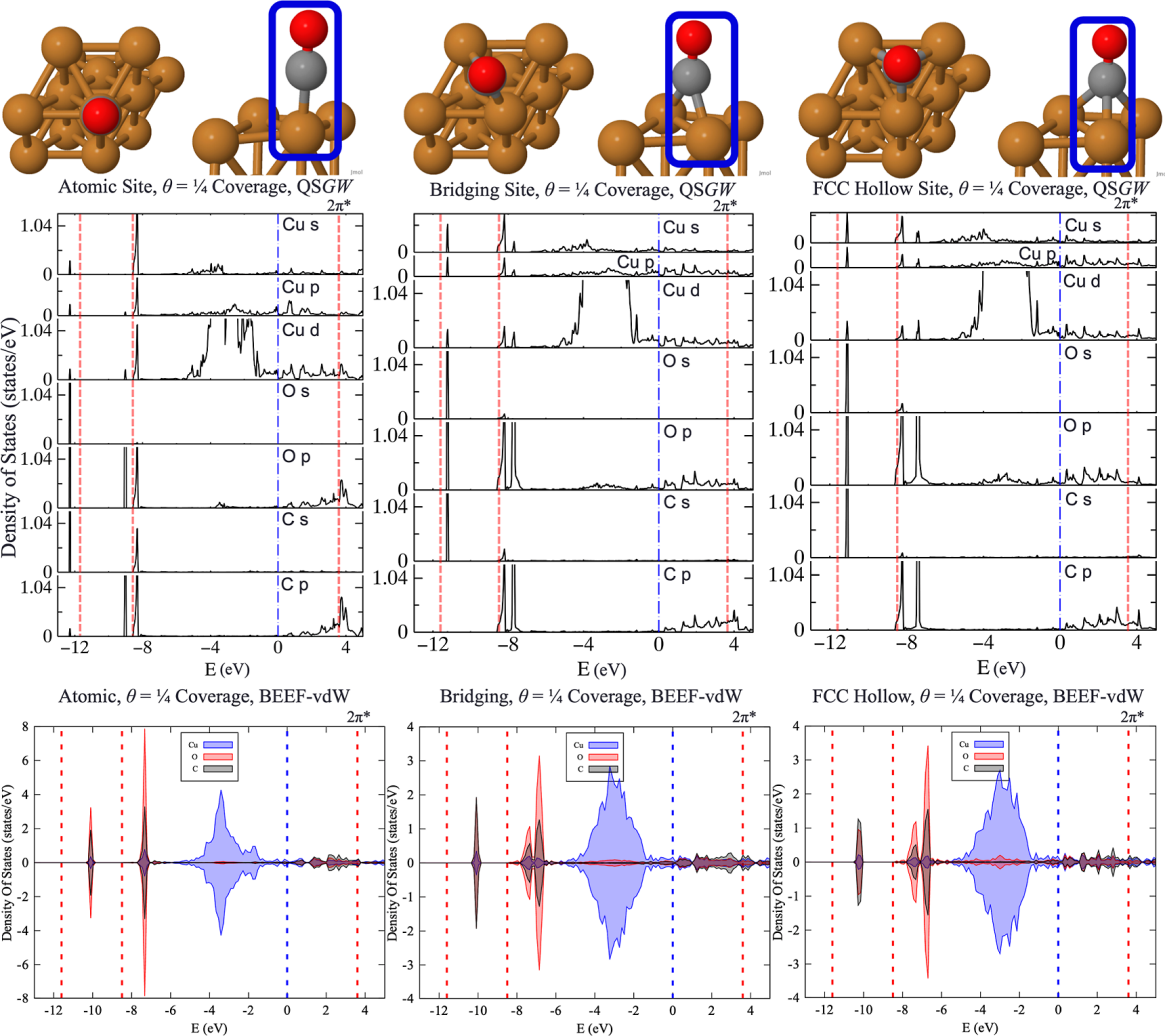
Theoretical PDOS, with dotted-red lines overlaid that reflect the molecular levels extracted from PE data. (**TOP**) Converged QS*GW* results from Questaal; (**BOTTOM**) DFT results from VASP. Dotted-red lines below *E*_F_ taken from He II-ultraviolet photoelectron data by Conrad et al.^16^ (*E*_F_ – 8.5 eV and *E*_F_ – 11.6 eV for θ = 1/3) and the 2π* LUMO from two-photon PE spectra by Wolf et al.^18^ (*E*_F_ 3.6 eV for θ = 1/3). Adapted from ref (51) and available under a CC-BY 4.0 license. Copyright 2024 Ha et al.

He II-ultraviolet photoelectron spectroscopy of CO on Cu(111) yields two peaks at *E*_F_ – 8.5 eV and *E*_F_ – 11.6 eV, at a coverage of θ = 1/3 (Figure 6).^13,21^ According to these reports, CO primarily resides on the atomic site, with some bridging sites occupied. The experiment cannot readily resolve which peak corresponds to σ- and which to π-character; therefore, Figure 6 does not label them. Nevertheless, the similarity with Pt(111) in orbital decomposition of the DOS indicates the CO levels order in the same way. Indeed, in most respects, the Pt and Cu cases are very similar: the peaks at *E*_F_ – 8.5 and *E*_F_ – 11.6 eV most likely originate from a confluence of contributions of atomic and bridging/hollow sites (Figure 6). This can be seen by the way the theoretical peaks straddle the PE data, depending on which site CO resides. Further, the position of the unoccupied 2π* peak depends on coverage (3.6 eV^18^ for θ = 1/3; and 3.35 eV^17^ for θ = 1/2). For θ = 1/2, this has been attributed to an estimated occupation ratio of 13:12 atomic:bridging sites.^29^ There is one important contrast between Pt and Cu cases: the 2π* LUMO has a moderately sharp peak only for the atomic site (2π* at 3.6 eV, close to experiment for lower coverages). For the other sites, the level broadens into a band that is approximately 4 eV wide.

This is likely because for the atomic site the C 2p_*x*_, O 2p_*x*_, and Cu 3d_*xz*_ form a linear chain of orbitals with π character that does not occur for the other geometries, which provides a more favorable environment for directional molecular bonds. Comparison to DFT may be found in Figure 6**(BOTTOM)**, where we display the PDOS of these three geometries from the DFT BEEF-vdW calculations in VASP. In keeping with the results found for CO on Pt(111) in Figure 2**(BOTTOM)**, the valence peaks are shifted by 1–2 eV from the experiment. In contrast, both QS**GW** and DFT provide broad, delocalized PDOS above the Fermi energy for the bridging and hollow site. QS**GW** provides resolution for a distinct peak, matching experiment, for the atomic site at *E*_F_ + 3.6 eV (Figure 6—**TOP**), but DFT exhibits broad bands for this site similar to those displayed for the bridging and hollow site (Figure 6—**BOTTOM**).

**Figure 7:**
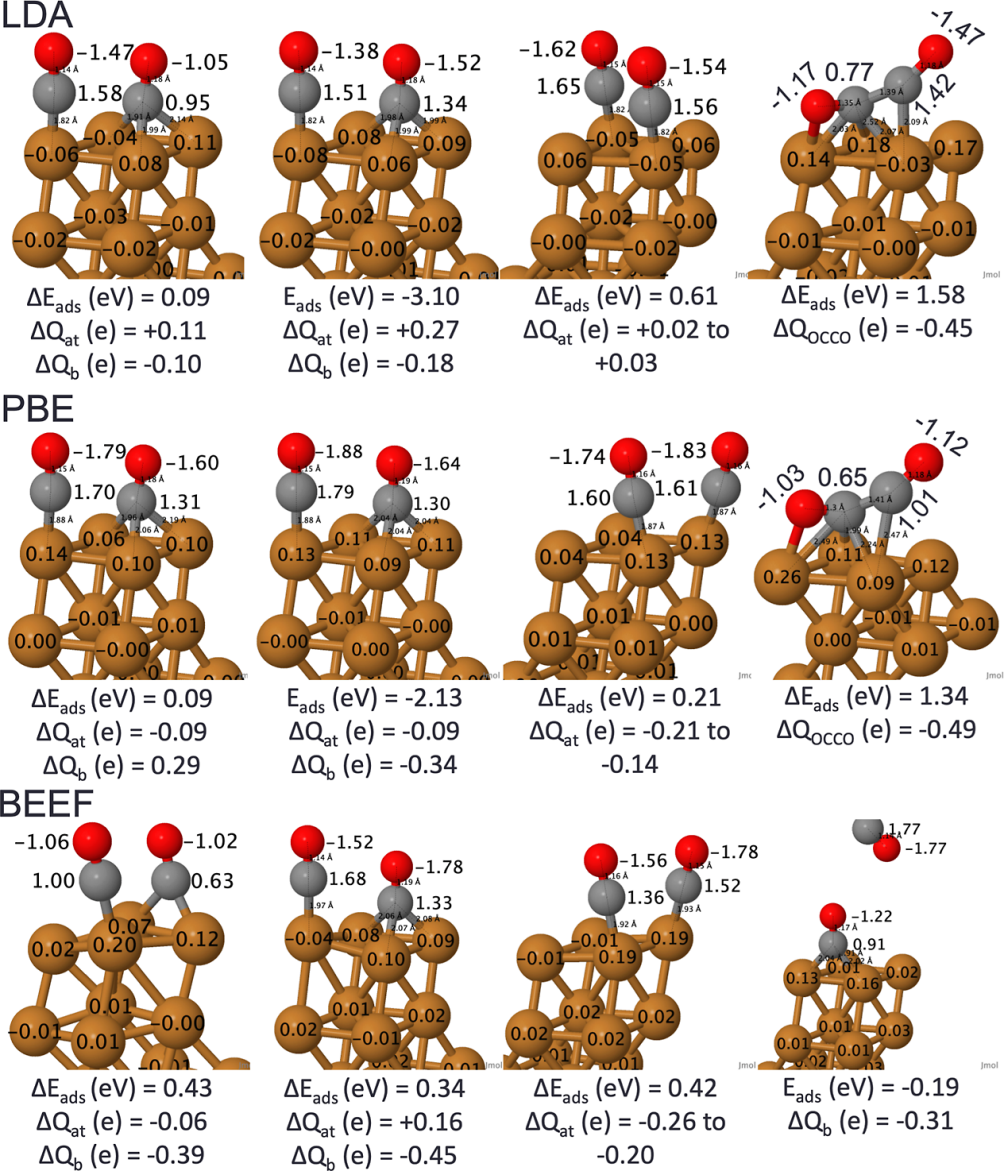
Visualization of 2CO/Cu(111) of atomic + bridging, atomic + fcc hollow, the OCCO dimer with adsorption energies (*E*_ads_, eV), relative adsorption energies (**Δ***E*_ads_, eV), Bader charges (**Δ***Q*, e), and bond lengths (Å, Supporting Information Table S4). Cu—dark orange, C—dark gray, O—red. Adapted from ref (51) and available under a CC-BY 4.0 license. Copyright 2024 Ha et al.

At higher coverages, θ = 1/2, we consider CO coadsorbed on the atomic + bridging sites, atomic + fcc hollow sites, and atomic + atomic sites (Figure 7). At θ = 1/2 coverage, the BEEF-vdW functional predicts a binding energy of +0.24 eV (atomic + bridging) as compared to −0.13 eV at θ = 1/4, weakening the binding compared to experiment’s,^58^ whereas LDA and PBE overbind the adsorption energies. BEEF-vdW adsorbed CO weaker than the experiment; the ramifications of this result in the neighboring CO desorbing from the surface for the global minimum. Moreover, the LDA and PBE functionals often relaxed the bridging site lower along the *z*-axis to resemble the hollow site. Bader charge analysis reveals that CO is slightly electronegative relative to Cu. C–O bond lengths remained similar when relaxed under the different functionals, with Cu–C bonds exhibiting a greater range (1.87–1.97 Å for PBE’s atomic site; 1.92–1.97 Å for BEEF-vdW), probably originating from steric interactions between neighboring CO molecules. While the OCCO dimer was easily found on Pt(111), it was difficult to stabilize on Cu(111). This OCCO dimer is stable only for LDA and PBE functionals; in the BEEF-vdW case, it would instead relax to coadsorbed atomic + atomic sites. However, this may be in keeping with recent experimental-theoretical studies on the OCCO dimer on various facets of Cu.^57,60^ In situ Raman spectroscopy combined with DFT calculations have attributed the OCCO dimer to be a key reaction intermediate in the selectivity of C_2_ products, and it may explain the unique reactivity of Cu(110) over Cu(111).^57^ Similarly, Fourier transform infrared spectroscopy detected the presence of the hydrogenated CO dimer on Cu(100) and not on Cu(111), highlighting that this may also explain Cu(100)’s high reactivity compared to Cu(111).^60^

**Figure 8:**
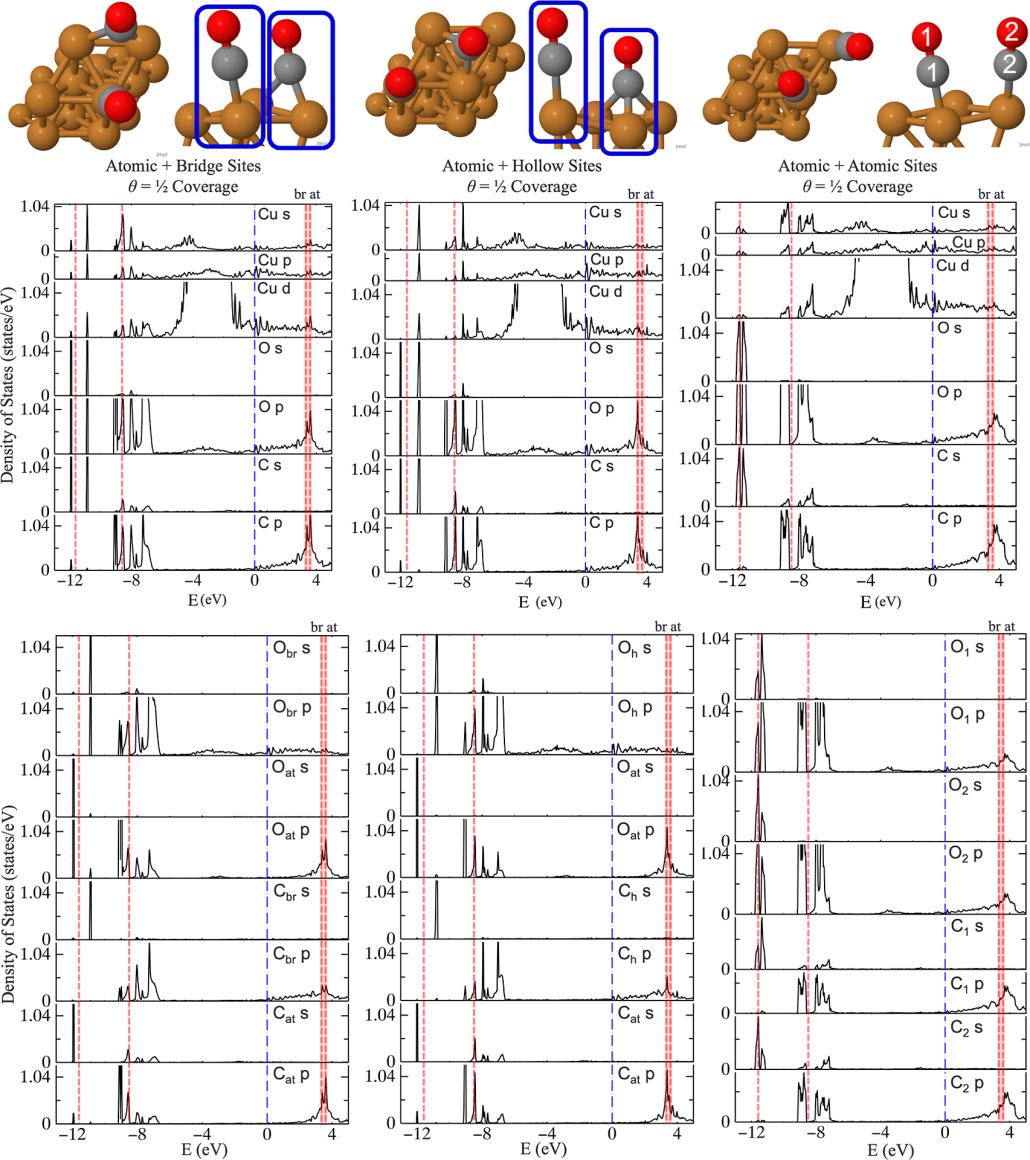
PDOS of 2CO/Cu(111) from QS*GW* calculations in geometries generated by BEEF-vdW. (**TOP**) Elemental analysis of Cu, C, and O orbital projections from the Cu–C–O atoms outlined in blue; (**BOTTOM**) atomic contributions of each C and O with subscripts indicating the specific atom to orbital contribution at—atomic, br—bridging, and h—hollow, and for coadsorbed atomic + atomic COs, numerical subscripts 1 and 2 were utilized to differentiate C and O atoms. Dotted-red lines below *E*_F_ taken from He II-ultraviolet photoelectron data by Conrad et al.^16^ (*E*_F_ – 8.5 eV and *E*_F_ – 11.6 eV) and the 2π* LUMO from two-photon PE spectra by Wolf et al.^18^ (*E*_F_ 3.6 eV for θ = 1/3) and Hertel et al.^17^ (*E*_F_ 3.35 eV for θ = 1/2). Adapted from ref (51) and available under a CC-BY 4.0 license. Copyright 2024 Ha et al.

Photoelectron spectroscopy of ref (16) was measured for the relatively low coverage of θ = 1/3, but by comparing θ = 1/4 to θ = 1/2, we can probe CO–CO interactions. Theory predicts two peaks straddling the observed *E*_F_ – 11.6 peak. Similarly, theory predicts the *E*_F_ – 8.5 peak to consist of multiple states straddling the observed level (see Figure 8, **TOP**). For both peaks, the average of the theoretical peaks is slightly higher than the experiment and offset slightly from the theoretical average of θ = 1/4 coverages. If the theory is a reliable predictor of these levels, this offset may be interpreted in part as the experimental consisting of an asymmetric occupation of (majority) atomic sites and (minority) bridging sites. Moreover, the offset is a signature of CO–CO interactions. It appears to be a steric effect: the CO molecules are slightly angled away from each other.

In order to examine in detail the CO–CO interactions, Figure 8**(BOTTOM)** displays the s- and p-orbital contributions from each C and O of these coadsorbed CO molecules on Cu(111). In contrast to the OCCO dimer in Figure 4 on Pt(111), which displayed identical spectra for C and O atoms, the coadsorbed atomic + atomic CO molecules exhibit inverse symmetry to one another near the deeper, experimental peak of *E*_F_ – 11.6 eV. Namely, C_1_’s s- and O_1_’s s-, p-orbital contributions display a more significant peak to the right of the experimental value, whereas C_2_’s s- and O_2_’s s-, p-orbital contributions feature a more prominent peak to the left of the experimental value. Contributions to the *E*_F_ – 8.5 eV may result from p-orbital contributions from any of the coadsorbed atomic + (atomic, bridging, and hollow) CO molecules. In comparison to the coadsorbed CO molecules on Pt(111), where the orbital projections from the atop CO dominate and shift due to the influence of the neighboring CO molecule, p-orbital resonance from atomic, bridging, and hollow CO atoms on Cu(111) can occur. Specifically, these p-dominant resonances feature at *E*_F_ – 8.5 and *E*_F_ + 3.6 eV (attributed by the experiment to majority atop CO molecules)^18^ and *E*_F_ + 3.35 eV (with a suggested occupation of 13:12 atomic:bridging sites).^17,29^

As previously noted, two-photon spectra was taken at both θ = 1/3 coverage^18^ and θ = 1/2 coverage^17^ for the LUMO, where the peak shifts from *E*_F_ + 3.6 to *E*_F_ + 3.35 eV. This provides another opportunity to benchmark the theory, which indeed predicts a similar coverage dependence (compare Figures 6–8). Rather unexpectedly, the peaks become sharper for all three geometries due to resonance of the p-orbital contributions of C and O atoms at these different sites. This showcases that CO–CO interactions can be unique, depending on the TM surface with this study showcasing the differences between Pt(111) and Cu(111). Taking experiment and theory together, we can conclude the following: (1) at low coverage, CO predominantly resides on the atomic site; (2) at higher coverages, the atomic site is still dominant but less so; (3) the spectroscopy is modified by CO–CO interactions via coupling of adjacent molecules, which can be detected for both occupied states and the LUMO. Conclusion (3) is new, while experimental findings have also drawn conclusions (1) and (2), albeit imprecisely. Finally (4) binding of CO pairs into an OCCO molecule appears to have a marked effect on the spectroscopy but may not be energetically favorable, based on (admittedly unreliable) DFT theory.

Correlating site positions to PE data can be useful in other contexts. Other low-index facets of Cu(100) and Cu(110) and higher-index facets such as Cu(711) and Cu(210) are often considered highly reactive for specific CO_2_ products.^5^ Cu(110) and Cu(210) exhibit Faradaic efficiencies of ca. 50% or higher for methane production; Cu(711) and Cu(100) in contrast hover at ca. 40% or higher for ethylene production.^5^ Both low-index and higher-index facets feature different atomic packing than (111); (110) and higher index facets often feature bridging, kinked, or terraced facets, whereas (100) is planar but less compact than (111). Greater characterization and accurate prediction of the properties of these interfaces can identify and inform the spectra and selectivity observed in the experiment.

## 4 Conclusions

In our QS**GW** study of CO on Pt(111) and Cu(111), we were able to resolve key spectral features of deeper states below the Fermi energy and qualify contributions of the CO site to the LUMO 2π* at various coverages. While previous studies could partially capture some of the states, they often utilized HSE or DFT + *U* methods, which require manipulation of either Hartree–Fock exchange in HSE or the *U* value in DFT + *U* in order to match the experiment. Essentially, an answer must be known beforehand to set adjustable parameters. QS**GW**-level calculations appear to accurately explain available observations of CO on Pt(111) and Cu(111) without model assumptions or free parameters, including detailed spectral features from photoelectron and PE experiments. Resolving the puzzle of CO adsorption on transition metals required both self-consistent *GW*-level calculations and an interfacial model that included the permutations of sites and coverages that may be possibly realized. Since CO is a key reaction intermediate and product in CO_2_ reduction to valuable C_2+_ products, interest remains high in increasing the selectivity and activity through electrochemistry and plasmonic means of catalysis.^5,22,23^ This study acts as a stepping stone to a comprehensive understanding of CO_2_ reduction. Self-consistent *GW* calculations can potentially elucidate other CO-related intermediates key to the reduction process, identifying spectral features related to the facet dependence of reactivity. Looking forward, RPA total energy from LDA-based eigenfunctions seem to significantly improve energetics relative to common DFT functionals for these systems.^25^ Since that work constructed the total energy from DFT eigenfunctions, which from the Bader analysis we cannot expect to be reliable, the RPA total energy from QS**GW** should be significantly better. In QS**GW**, the path of adiabatic connection bridging the noninteracting Hamiltonian to the interacting one is better than the path given by Kohn–Sham theory, by its construction.^10^ Thus QS**GW**-based RPA total energy should be another powerful tool to resolve and predict reaction enthalpies.

## Supporting Information Available

Theoretical bond lengths as compared to the experiment, computing parameters and time on Perlmutter for a subset of calculations, QS**GW** calculations without and with spin–orbit coupling for atop CO on Pt(111), and elemental PDOS for DFT results (PDF)

## Abbreviations

QS**GW**: quasiparticle self-consistent *GW*
PW-DFT: plane-wave density functional theory
LDA: local density approximation
HOMO: highest occupied molecular orbital
LUMO: least unoccupied molecular orbital
IRAS: infrared-absorption spectroscopy
STM: scanning tunneling microscopy

## References

[ref1] Lamberti C.; Zecchina A.; Groppo E.; Bordiga S. Probing the surfaces of heterogeneous catalysts by in situ IR spectroscopy. Chem. Soc. Rev. 2010, 39 (12), 4951–5001. 10.1039/c0cs00117a. 21038053

[ref2] Schmidt T.; Gasteiger H.; Behm R. Rotating disk electrode measurements on the CO tolerance of a high-surface area Pt/vulcan carbon fuel cell catalyst. J. Electrochem. Soc. 1999, 146 (4), 1296 10.1149/1.1391761.

[ref3] Mayrhofer K.; Strmcnik D.; Blizanac B.; Stamenkovic V.; Arenz M.; Markovic N. Measurement of oxygen reduction activities via the rotating disc electrode method: From Pt model surfaces to carbon-supported high surface area catalysts. Electrochim. Acta 2008, 53 (7), 3181–3188. 10.1016/j.electacta.2007.11.057.

[ref4] Schmidt T. J.; Gasteiger H. A.; Stäb G. D.; Urban P. M.; Kolb D. M.; Behm R. J. Characterization of High-Surface-Area Electrocatalysts Using a Rotating Disk Electrode Configuration. J. Electrochem. Soc. 1998, 145 (7), 2354 10.1149/1.1838642.

[ref5] Nitopi S.; Bertheussen E.; Scott S. B.; Liu X.; Engstfeld A. K.; Horch S.; Seger B.; Stephens I. E.; Chan K.; Hahn C.; et al. Progress and perspectives of electrochemical CO2 reduction on copper in aqueous electrolyte. Chem. Rev. 2019, 119 (12), 7610–7672. 10.1021/acs.chemrev.8b00705. 31117420

[ref6] Qiao J.; Liu Y.; Hong F.; Zhang J. A review of catalysts for the electroreduction of carbon dioxide to produce low-carbon fuels. Chem. Soc. Rev. 2014, 43 (2), 631–675. 10.1039/C3CS60323G. 24186433

[ref7] Patra A.; Peng H.; Sun J.; Perdew J. P. Rethinking CO adsorption on transition-metal surfaces: Effect of density-driven self-interaction errors. Phys. Rev. B 2019, 100 (3), 035442 10.1103/PhysRevB.100.035442.

[ref8] van Schilfgaarde M.; Kotani T.; Faleev S. Quasiparticle self-consistent g w theory. Phys. Rev. Lett. 2006, 96 (22), 226402 10.1103/PhysRevLett.96.226402. 16803332

[ref9] Sponza L.; Pisanti P.; Vishina A.; Pashov D.; Weber C.; Van Schilfgaarde M.; Acharya S.; Vidal J.; Kotliar G. Self-energies in itinerant magnets: a focus on Fe and Ni. Phys. Rev. B 2017, 95 (4), 041112 10.1103/PhysRevB.95.041112.

[ref10] Kotani T.; Van Schilfgaarde M.; Faleev S. V. Quasiparticle self-consistent G W method: A basis for the independent-particle approximation. Phys. Rev. B: Condens. Matter Mater. Phys. 2007, 76 (16), 165106 10.1103/PhysRevB.76.165106.

[ref11] Caruso F.; Dauth M.; Van Setten M. J.; Rinke P. Benchmark of *GW* approaches for the *GW* 100 test set. J. Chem. Theory Comput. 2016, 12 (10), 5076–5087. 10.1021/acs.jctc.6b00774. 27631585

[ref12] Faleev S. V.; Mryasov O. N.; van Schilfgaarde M. Effect of correlations on electronic structure and transport across (001) Fe/MgO/Fe junctions. Phys. Rev. B: Condens. Matter Mater. Phys. 2012, 85 (17), 174433 10.1103/PhysRevB.85.174433.

[ref13] Ismail-Beigi S. Justifying quasiparticle self-consistent schemes via gradient optimization in Baym–Kadanoff theory. J. Phys.: Condens. Matter 2017, 29 (38), 385501 10.1088/1361-648X/aa7803. 28593935

[ref14] Cunningham B.; Grüning M.; Pashov D.; Van Schilfgaarde M. QS *GW* ^: Quasiparticle self-consistent *GW* with ladder diagrams in W. Phys. Rev. B 2023, 108 (16), 165104 10.1103/PhysRevB.108.165104.

[ref15] Pashov D.; Acharya S.; Lambrecht W. R.; Jackson J.; Belashchenko K. D.; Chantis A.; Jamet F.; van Schilfgaarde M. Questaal: A package of electronic structure methods based on the linear muffin-tin orbital technique. Comput. Phys. Commun. 2020, 249, 107065 10.1016/j.cpc.2019.107065.

[ref16] Conrad H.; Ertl G.; Küppers J.; Latta E. Photoelectron spectroscopy from CO adsorbed on a Cu (111) surface. Solid State Commun. 1975, 17 (5), 613–616. 10.1016/0038-1098(75)90547-5.

[ref17] Hertel T.; Knoesel E.; Hasselbrink E.; Wolf M.; Ertl G. Unoccupied adsorbate states of COCu (111) analyzed with two-photon photoemission. Surf. Sci. 1994, 317 (3), L1147–L1151. 10.1016/0039-6028(94)90282-8.

[ref18] Wolf M.; Hotzel A.; Knoesel E.; Velic D. Direct and indirect excitation mechanisms in two-photon photoemission spectroscopy of Cu (111) and CO/Cu (111). Phys. Rev. B: Condens. Matter Mater. Phys. 1999, 59 (8), 5926 10.1103/PhysRevB.59.5926.

[ref19] Tsilimis G.; Kutzner J.; Zacharias H. Photoemission study of clean and c (4× 2)-2CO-covered Pt (111) using high-harmonic radiation. Appl. Phys. A: Mater. Sci. Process. 2003, 76, 743–749. 10.1007/s00339-002-1496-3.

[ref20] Anazawa T.; Kinoshita I.; Matsumoto Y. Two-photon photoemission study ofCO/Pt (111). J. Electron Spectrosc. Relat. Phenom. 1998, 88, 585–590. 10.1016/S0368-2048(97)00131-X.

[ref21] Feibelman P. J.; Hammer B.; Nørskov J. K.; Wagner F.; Scheffler M.; Stumpf R.; Watwe R.; Dumesic J. The CO/Pt(111) Puzzle. J. Phys. Chem. B 2001, 105 (18), 4018–4025. 10.1021/jp002302t.

[ref22] Mahapatra S.; Schultz J. F.; Li L.; Zhang X.; Jiang N. Controlling Localized Plasmons via an Atomistic Approach: Attainment of Site-Selective Activation inside a Single Molecule. J. Am. Chem. Soc. 2022, 144 (5), 2051–2055. 10.1021/jacs.1c11547. 34978804

[ref23] Creel E. B.; Corson E. R.; Eichhorn J.; Kostecki R.; Urban J. J.; McCloskey B. D. Directing Selectivity of Electrochemical Carbon Dioxide Reduction Using Plasmonics. ACS Energy Lett. 2019, 4 (5), 1098–1105. 10.1021/acsenergylett.9b00515.

[ref24] Stroppa A.; Termentzidis K.; Paier J.; Kresse G.; Hafner J. CO adsorption on metal surfaces: A hybrid functional study with plane-wave basis set. Phys. Rev. B: Condens. Matter Mater. Phys. 2007, 76 (19), 195440 10.1103/PhysRevB.76.195440.

[ref25] Schimka L.; Harl J.; Stroppa A.; Grüneis A.; Marsman M.; Mittendorfer F.; Kresse G. Accurate surface and adsorption energies from many-body perturbation theory. Nat. Mater. 2010, 9 (9), 741–744. 10.1038/nmat2806. 20657589

[ref26] Lazić P.; Alaei M.; Atodiresei N.; Caciuc V.; Brako R.; Blügel S. Density functional theory with nonlocal correlation: A key to the solution of the CO adsorption puzzle. Phys. Rev. B: Condens. Matter Mater. Phys. 2010, 81 (4), 045401 10.1103/PhysRevB.81.045401.

[ref27] Gajdoš M.; Hafner J. CO adsorption on Cu (1 1 1) and Cu (0 0 1) surfaces: Improving site preference in DFT calculations. Surf. Sci. 2005, 590 (2–3), 117–126. 10.1016/j.susc.2005.04.047.

[ref28] Olsen R.; Philipsen P.; Baerends E. CO on Pt (111): A puzzle revisited. J. Chem. Phys. 2003, 119 (8), 4522–4528. 10.1063/1.1593629.

[ref29] Hayden B.; Kretzschmar K.; Bradshaw A. An infrared spectroscopic study of CO on Cu (111): the linear, bridging and physisorbed species. Surf. Sci. 1985, 155 (2–3), 553–566. 10.1016/0039-6028(85)90013-5.

[ref30] Ogletree D.; Van Hove M.; Somorjai G. LEED intensity analysis of the structures of clean Pt (111) and of CO adsorbed on Pt (111) in the c (4× 2) arrangement. Surf. Sci. 1986, 173 (2–3), 351–365. 10.1016/0039-6028(86)90195-0.

[ref31] Nekrylova J.; Harrison I. Single hop diffusion of CO from bridge to top sites on Pt (111). J. Chem. Phys. 1994, 101 (2), 1730–1733. 10.1063/1.468435.

[ref32] Nekrylova J.; Harrison I. Site resolved adsorption dynamics of CO on Pt (111). Chem. Phys. 1996, 205 (1–2), 37–46. 10.1016/0301-0104(95)00304-5.

[ref33] Nekrylova J.; French C.; Artsyukhovich A.; Ukraintsev V.; Harrison I. Low temperature adsorption of CO on Pt (111): disequilibrium and the occupation of three-fold hollow sites. Surf. Sci. Lett. 1993, 295 (1–2), L987–L992. 10.1016/0167-2584(93)91008-c.

[ref34] Villegas I.; Weaver M. J. Carbon monoxide adlayer structures on platinum (111) electrodes: A synergy between in-situ scanning tunneling microscopy and infrared spectroscopy. J. Chem. Phys. 1994, 101 (2), 1648–1660. 10.1063/1.467786.

[ref35] Yang H. J.; Minato T.; Kawai M.; Kim Y. STM Investigation of CO ordering on Pt (111): From an isolated molecule to high-coverage superstructures. J. Phys. Chem. C 2013, 117 (32), 16429–16437. 10.1021/jp404231t.

[ref36] Wyckoff R. W. G. Crystal Structures; Wiley, 1963.

[ref37] Hoffmann R. A chemical and theoretical way to look at bonding on surfaces. Rev. Mod. Phys. 1988, 60 (3), 601 10.1103/RevModPhys.60.601.

[ref38] Yu M.; Trinkle D. R. Accurate and efficient algorithm for Bader charge integration. J. Chem. Phys. 2011, 134 (6), 064111 10.1063/1.3553716. 21322665

[ref39] Tang W.; Sanville E.; Henkelman G. A grid-based Bader analysis algorithm without lattice bias. J. Phys.: Condens. Matter 2009, 21 (8), 084204 10.1088/0953-8984/21/8/084204. 21817356

[ref40] Sanville E.; Kenny S. D.; Smith R.; Henkelman G. Improved grid-based algorithm for Bader charge allocation. J. Comput. Chem. 2007, 28 (5), 899–908. 10.1002/jcc.20575. 17238168

[ref41] Henkelman G.; Arnaldsson A.; Jónsson H. A fast and robust algorithm for Bader decomposition of charge density. Comput. Mater. Sci. 2006, 36 (3), 354–360. 10.1016/j.commatsci.2005.04.010.

[ref42] Kresse G.; Hafner J. Ab initio molecular dynamics for liquid metals. Phys. Rev. B: Condens. Matter Mater. Phys. 1993, 47 (1), 558 10.1103/PhysRevB.47.558. 10004490

[ref43] Kresse G.; Hafner J. Ab initio molecular-dynamics simulation of the liquid-metal–amorphous-semiconductor transition in germanium. Phys. Rev. B: Condens. Matter Mater. Phys. 1994, 49 (20), 14251 10.1103/PhysRevB.49.14251. 10010505

[ref44] Kresse G.; Furthmüller J. Efficiency of ab-initio total energy calculations for metals and semiconductors using a plane-wave basis set. Comput. Mater. Sci. 1996, 6 (1), 15–50. 10.1016/0927-0256(96)00008-0.

[ref45] Kresse G.; Furthmüller J. Efficient iterative schemes for ab initio total-energy calculations using a plane-wave basis set. Phys. Rev. B: Condens. Matter Mater. Phys. 1996, 54 (16), 11169 10.1103/PhysRevB.54.11169. 9984901

[ref46] Kohn W.; Sham L. J. Self-Consistent Equations Including Exchange and Correlation Effects. Phys. Rev. 1965, 140 (4A), A1133–A1138. 10.1103/PhysRev.140.A1133.

[ref47] Perdew J. P.; Burke K.; Ernzerhof M. Generalized Gradient Approximation Made Simple. Phys. Rev. Lett. 1996, 77 (18), 3865–3868. 10.1103/PhysRevLett.77.3865. 10062328

[ref48] Wellendorff J.; Lundgaard K. T.; Møgelhøj A.; Petzold V.; Landis D. D.; Nørskov J. K.; Bligaard T.; Jacobsen K. W. Density functionals for surface science: Exchange-correlation model development with Bayesian error estimation. Phys. Rev. B: Condens. Matter Mater. Phys. 2012, 85 (23), 235149 10.1103/PhysRevB.85.235149.

[ref49] Vidal J.; Botti S.; Olsson P.; Guillemoles J.-F.; Reining L. Strong Interplay between Structure and Electronic Properties in CuIn (S, Se) 2:<? format?> A First-Principles Study. Phys. Rev. Lett. 2010, 104 (5), 056401 10.1103/PhysRevLett.104.056401. 20366776

[ref50] Acharya S.; Pashov D.; Rudenko A. N.; Rösner M.; van Schilfgaarde M.; Katsnelson M. I. Importance of charge self-consistency in first-principles description of strongly correlated systems. npj Comput. Mater. 2021, 7 (1), 208 10.1038/s41524-021-00676-5.

[ref51] Ha M.-A.; Pashov D.; van shilfgaarde M. Correlating Optical and Structural Properties of CO on Transition Metal Surfaces. ChemRxiv 2024, 10.26434/chemrxiv-2024-0kn9q. PMC11912529 40103659

[ref52] Poelsema B.; Palmer R. L.; Comsa G. A thermal He scattering study of CO adsorption on Pt (111). Surf. Sci. 1984, 136 (1), 1–14. 10.1016/0039-6028(84)90651-4.

[ref53] Ertl G.; Neumann M.; Streit K. Chemisorption of CO on the Pt (111) surface. Surf. Sci. 1977, 64 (2), 393–410. 10.1016/0039-6028(77)90052-8.

[ref54] Schweizer E.; Persson B.; Tüshaus M.; Hoge D.; Bradshaw A. The potential energy surface, vibrational phase relaxation and the order-disorder transition in the adsorption system Pt {111}-CO. Surf. Sci. 1989, 213 (1), 49–89. 10.1016/0039-6028(89)90252-5.

[ref55] Azarhoosh P.; McKechnie S.; Frost J. M.; Walsh A.; Van Schilfgaarde M. Research Update: Relativistic origin of slow electron-hole recombination in hybrid halide perovskite solar cells. APL Mater. 2016, 4 (9), 091501 10.1063/1.4955028.

[ref56] Vandichel M.; Grönbeck H. A dimer path for CO dissociation on PtSn. Catal. Sci. Technol. 2019, 9 (3), 695–701. 10.1039/C8CY01989D.

[ref57] Zhao Y.; Zhang X.-G.; Bodappa N.; Yang W.-M.; Liang Q.; Radjenovica P. M.; Wang Y.-H.; Zhang Y.-J.; Dong J.-C.; Tian Z.-Q.; et al. Elucidating electrochemical CO 2 reduction reaction processes on Cu (hkl) single-crystal surfaces by in situ Raman spectroscopy. Energy Environ. Sci. 2022, 15 (9), 3968–3977. 10.1039/d2ee01334g.

[ref58] Hollins P.; Pritchard J. Interactions of CO molecules adsorbed on Cu (111). Surf. Sci. 1979, 89 (1–3), 486–495. 10.1016/0039-6028(79)90633-2.

[ref59] Moler E. J.; Kellar S. A.; Huff W.; Hussain Z.; Chen Y.; Shirley D. A. Spatial structure determination of (√ 3×√ 3) R30° and (1.5× 1.5) R18° CO or Cu (111) using angle-resolved photoemission extended fine structure. Phys. Rev. B: Condens. Matter Mater. Phys. 1996, 54 (15), 10862 10.1103/PhysRevB.54.10862. 9984884

[ref60] Pérez-Gallent E.; Figueiredo M. C.; Calle-Vallejo F.; Koper M. T. Spectroscopic observation of a hydrogenated CO dimer intermediate during CO reduction on Cu (100) electrodes. Angew. Chem., Int. Ed. 2017, 56 (13), 3621–3624. 10.1002/anie.201700580. 28230297

