# Supplementary material for: Correlating Optical and Structural Properties of CO on Transition Metal Surfaces
Source: J Phys Chem C Nanomater Interfaces. 2025 Mar 3;129(10):4923–36. doi: 10.1021/acs.jpcc.4c07418 (PMC11912529; doi:10.1021/acs.jpcc.4c07418)
Supplement: Supplementary file 1 — jp4c07418_si_001.pdf [file jp4c07418_si_001.pdf]

Correlating Optical and Structural Properties of CO on Transition Metal Surfaces

Mai-Anh Ha,[†] Dimitar Pashov,[‡] and Mark van Schilfgaarde[§*]

[†] Computational Science Center, National Renewable Energy Laboratory, 15013 Denver West Parkway, Golden, Colorado 80401, United States,

[‡] Department of Physics, King's College London, Strand, London, WC2R 2LS, United Kingdom

[§] Materials Chemical & Computational Science, National Renewable Energy Laboratory, 15013 Denver West Parkway, Golden, Colorado 80401, United States

Supporting Information

Table S1. Bonding Characteristics of CO Adsorbed on Pt (111) at Various Sites at θ = ¼

Bonds	Exp r(Å)	LDA r(Å)	PBE r(Å)	BEEF-vdW r(Å)
C-O	1.15±0.05[1]	1.15 (at),<br>1.17 (br),<br>1.19 (h)	1.16 (at),<br>1.18 (br),<br>1.19 (h)	1.16 (at),<br>1.18 (br),<br>1.19 (h)
Pt-C (atomic)	1.85±010[1]	1.82	1.84	1.84
Pt-C (bridging)	2.08±0.07[1]	2.00	2.02	2.02
Pt-C (hollow)		2.08	2.11	2.12

Table S2. Bonding Characteristics of 2CO Co-Adsorbed at Various Sites on Pt (111) at θ = ½

Bonds	Exp r(Å)	LDA r(Å)	PBE r(Å)	BEEF r(Å)
C-O	1.15±0.05[1]	1.15 (at); 1.18 (b)<br>1.15 (at); 1.19 (h)<br>1.19 (OCCO)	1.15 (at); 1.18 (b)<br>1.15 (at); 1.19 (h),<br>1.19 (OCCO)	1.15 (at) ;1.18 (b)<br>1.15 (at); 1.19 (h)<br>1.19 (OCCO)
Pt-C (atomic)	1.85±0.01[1]	1.83	1.86	1.86
Pt-C (bridging)	2.08±0.07[1]	1.97-2.02	2.02-2.04	2.01-2.03
Pt-C (hollow)		2.08	2.11	2.11

Table S3. Bonding Characteristics of CO Adsorbed at Various Sites on Cu (111) at θ = ¼

Bonds	Exp r(Å)	LDA r(Å)	PBE r(Å)	BEEF-vdW r(Å)
C-O		1.15 (at),<br>1.17 (br),<br>1.18 (h)	1.16 (at),<br>1.18 (br),<br>1.18 (h)	1.16 (at),<br>1.17 (br),<br>1.18 (h)

Cu-C (atomic)	1.91[1]	1.80	1.85	1.91
Cu-C (bridging)		1.93	1.98	2.03
Cu-C (hollow)		2.00	2.04	2.09

Table S4. Bonding Characteristics of CO Adsorbed at Various Sites on Cu (111) at θ = ½

Bonds	Exp r(Å)	LDA r(Å)	PBE r(Å)	BEEF-vdW r(Å)
C-O		1.14 (at) + 1.18 (b),<br>1.14 (at) + 1.18 (h),<br>1.15 (at) + 1.15 (at),<br>1.18-1.35 (OCCO)	1.15 (at) + 1.18 (b),<br>1.14 (at) + 1.19 (h),<br>1.16 (at) + 1.16 (at),<br>1.18-1.30 (OCCO)	1.15 (at) + 1.18 (b),<br>1.14 (at) + 1.19 (h),<br>1.16 (at) + 1.15 (at),<br>1.17 (b) + 1.14 (desorb)
Cu-C (atomic)	1.91[2]	1.82	1.87-1.97	1.92-1.97
Cu-C (bridging)		1.91-1.99	1.96-2.06	1.94-2.13
Cu-C (hollow)		1.98-2.01	2.02-2.07	2.06-2.08

Table S5. Computing Time on Perlmutter

System	# of Atoms	Computing Time (hours)	# of Nodes
Atomic CO on Pt (111)	18	17.64	81
Bridging CO on Pt (111)	18	3.97	81
Hollow CO on Pt (111)	18	4.52	81
Atomic CO + Bridging CO on Pt (111)	28	7.95	81
Atomic CO + Hollow CO on Pt (111)	28	4.42	81
OCCO on Pt (111)	28	10.01	81

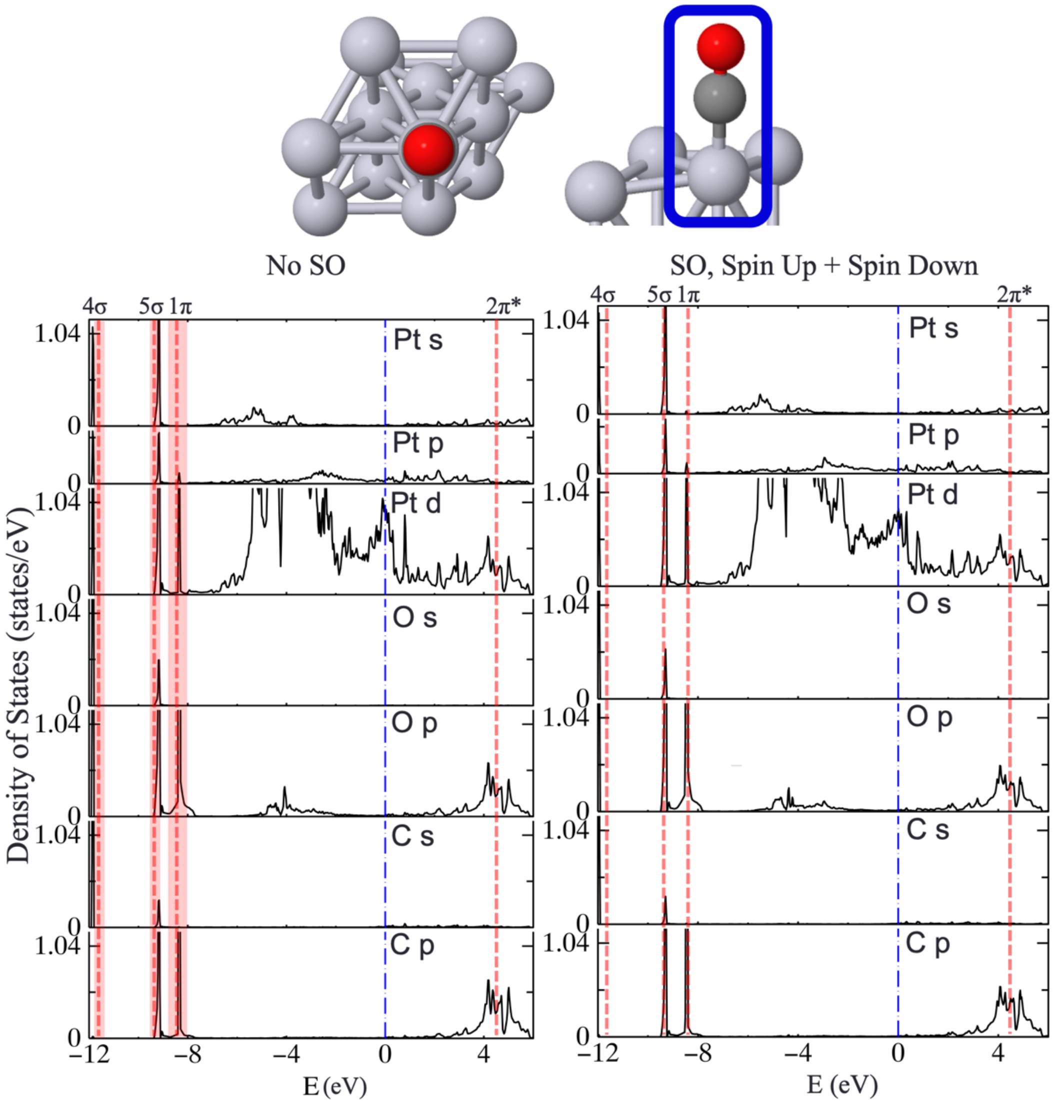

Figure S1. PDOS from Questaal calculations, comparing without and with spin-orbit (SO) coupling.

In our present calculations and figures in the main text, spin-orbit coupling was not included. We have added to the SI a comparison of our original calculation and spin-orbit coupling with spin up + spin down added together, see **SI Figure 1**.

Bibliography

(1) Ogletree, D.; Van Hove, M.; Somorjai, G. LEED intensity analysis of the structures of clean Pt (111) and of CO adsorbed on Pt (111) in the c (4× 2) arrangement. *Surf. Sci.* **1986**, *173* (2-3), 351-365.
(2) Moler, E. J.; Kellar, S. A.; Huff, W.; Hussain, Z.; Chen, Y.; Shirley, D. A. Spatial structure determination of (√ 3×√ 3) R30° and (1.5× 1.5) R18° CO or Cu (111) using angle-resolved photoemission extended fine structure. *Phys. Rev. B* **1996**, *54* (15), 10862.
